# Primary Central Nervous System Lymphoma (PCNSL): Analysis of Treatment by Gamma Knife Radiosurgery and Chemotherapy in a Prospective, Observational Study

**DOI:** 10.7759/cureus.697

**Published:** 2016-07-18

**Authors:** Andres M Alvarez-Pinzon, Aizik L Wolf, Heather Swedberg, Sammie R Coy, Jose E Valerio

**Affiliations:** 1 Miami Neuroscience Center, Larkin Community Hospital; 2 Biotechnology - NeuroOncology, Biotechnology Advanced Academic Programs | Johns Hopkins University; 3 Larkin Community Hospital, St. George's University School of Medicine; 4 Miami Neuroscience Center, Larkin Community hospital

**Keywords:** primary cns lymphoma, radiosurgery, gamma knife, chemotherapy, brain tumor, methotrexate, lymphoma, pcnsl, gkrs

## Abstract

Background:

Primary central nervous system lymphoma (PCNSL) is a rare cancer accounting for less than 3% of primary brain and central nervous system (CNS) tumors. Tissues involved include the brain parenchyma, leptomeninges, eyes, and spinal cord. High-dose methotrexate (MTX) is the gold standard for newly diagnosed PCNSL. However, Gamma Knife radiosurgery (GKRS) may be efficacious as a co-adjuvant treatment. The purpose of this prospective observational cohort study is to determine the effectiveness of MTX in combination with GKRS in the treatment of PCNSL.

Methods:

This is a prospective, observational cohort study evaluating the treatment of histologically confirmed PCNSL with MTX as a single agent in a dose of 8 g/m2 (control) and treatment with MTX, plus GKRS. Strict inclusion and exclusion criteria were employed. Primary outcomes were measured by survival rate. Secondary outcomes were assessed by the tumor’s responsiveness to treatment and reduction in size as noted on imaging.

Results:

Between January 2007 and January 2012, 128 charts were evaluated. Included in this evaluation were 73 chemotherapy (control) and 55 chemotherapy, plus GKRS, patients (variable). The follow-up period was 24 to 49 months (mean: 36.9 months). There were no statistically significant differences in patient demographics or histology diagnosis. Patients were treated with GK doses ranging from 11 Gy to 16 Gy (median: 11 Gy). The median survival rate from initial diagnosis was 26.8 months in the chemotherapy group and 47.6 in the chemotherapy, plus GKRS, group (p-value: 0.0034). All lesions showed a complete response after GKRS when evaluated using magnetic resonance imaging after three to eight weeks (mean range: 6.3 weeks).

Conclusions:

The use of GKRS is non-invasive, safe, and shows rapid success, improving the prognosis of the patient. This noninvasive treatment modality should be considered as an option for patients with PCNSL. In our study, GKRS as a co-adjuvant therapy to high-dose methotrexate was statistically significant for greater tumor control, enhanced overall survival period, and a lesser number of complications.

## Introduction

Primary central nervous system lymphoma (PCNSL) is a rare cancer accounting for less than 3% of primary brain and central nervous system (CNS) tumors [1]. PCNSL routinely presents as a single or multifocal lesion capable of infiltrating the cortex, with extension into the white and/or gray matter. Areas of necrosis may be found, especially in the immunocompromised. The primary CNS extranodal high-grade malignant cells are often large or immunoblastic cells of B-cell non-Hodgkin's, originating from the brain parenchyma, spinal cord, leptomeninges, or eyes [2]. PCNSL is typically limited to the CNS, often as intra-axial nodules with diffuse extension throughout the meningeal, periventricular, and perivascular spaces.

The prognosis of untreated PCNSL remains grim with low median survival time; therefore, the most effective treatment should be initiated as soon as possible [3]. In comparison to other brain tumors, resection is not indicated for the treatment of newly diagnosed PCNSL; management with radiation and/or chemotherapy is recommended [4-5]. Gamma Knife radiosurgery (GKRS), also called stereotactic radiosurgery, is a non-invasive treatment that utilizes beams of gamma radiation to target a specific lesion. While GKRS has not yet been used universally in the treatment of the PCNSL, it is a relatively safe alternative, yielding rapid and successful results, and ultimately improving the prognosis and quality of life of the patient [6]. The role of radiation therapy for initial management of PCNSL, however, remains controversial. Clinical trials should attempt to improve the therapeutic index of this modality.

A major challenge in the management of patients with PCNSL remains the delivery of aggressive chemotherapeutic treatment with preservation of neurocognitive function. Routes of chemotherapy administration include intravenous, intraocular, intraventricular, or intra-arterial. Multiple trials have outlined different methotrexate-based chemotherapy regimens and are utilizing local techniques to improve drug delivery. A study of the blood–brain barrier (BBB) permeability in a rat brain tumor model established a large heterogeneity of microvascular leakage; the vasculature within and around the brain tumors has a wide range of permeability, from typical capillaries with no (BBB) leakage to tumor vasculature allowing free entry of large molecules [7]. High-dose MTX (> 1 g/m^2^) has been shown to be an independent factor correlating with survival. Thus, MTX is administered in high doses, up to 8 g/m^2^, in order to achieve therapeutic drug concentrations in the tumor and surrounding brain parenchyma [7]. Intravenous (IV) doses less than 0.5 g/m^2_,_^ similar to what has been used in the treatment of malignancies outside of the brain, reach CNS concentrations generally thought to be non-cytotoxic [7].

The purpose of this prospective observational cohort study is to determine the effectiveness of chemotherapy using MTX in doses of 8 g/m^2^  in combination with GKRS in the treatment of PCNSL.

## Materials and methods

This is a prospective observational cohort study evaluating the effect and survival rate of patients with a histological diagnosis of PCNSL treated with MTX alone (control) and MTX, plus GKRS (variable). Charts were reviewed between January 2007 and January 2012. The study received approval from the Larkin Community Hospital Institutional Review Board (approval #LCH - 022015). The IRB waived the informed consent process. A single experienced, blinded post-doctoral fellow used Research Electronic Data Capture (REDCap) to collect data from eligible patients. Eligible patients underwent GKRS and/or a chemotherapy protocol using MTX at the same institution. All patients included in the study were diagnosed with PCNSL by biopsy and pathohistology report of the brain lesion observed on MRI. Strict inclusion and exclusion criteria were employed, as outlined in Table 1.

**Table 1:** Inclusion and Exclusion Criteria

INCLUSION AND EXCLUSION CRITERIA	
Inclusion Criteria	Exclusion Criteria
• Patients with a diagnosis of PCNSL and treated by Methotrexate	• AIDS diagnosis at time of treatment and diffuse CNS lymphomas
• Primary CNS lymphoma diagnosed by neurohistology pathologist	• Patients with addiction to illegal drugs, solvents, or alcohol who are currently using or previously attempted or failed a treatment program
	• Patients with bacteremia, systemic infection, or infection
	• Patients pregnant or nursing
	• History of previous brain surgery or brain tumor
	• History of systemic disease

Primary inclusion criteria were subjects who received MTX therapy as a single agent in a standard protocol dosage of 8 g/m^2^. The presence and size of the tumor, as well as its localization, were confirmed using a brain MRI protocol. The maximal brain tumor was measured at 1 cm and 2 cm proximal to the most distal aspect of its midline. Primary outcomes were measured by survival rate. Secondary outcomes were assessed by the tumor’s responsiveness to treatment and reduction in size as noted on MRI and/or CT scan.

### Statistical analysis

A priori sample size calculation was performed on the basis of a prior study, which evaluated the survival rate in a sample size of 40 patients [8]. The study implied a sample size of at least 31 subjects per group is necessary to detect the minimal clinically important difference in the mortality rate of 1.5 standard deviations (σ = 1.5, α = 0.05, β = 0.20). Additionally, a general estimate for detecting a one-unit change on an ordinal scale of 136 (σ = 1.0, α = 0.05, β = 0.20) resulted in the same number. The Wilcoxon Rank-Sum Test was used for comparison of continuous data between the groups. Differences between means were analyzed using two-sided T-tests. Comparison of categorical data was performed using Pearson’s Chi-square test or Fisher’s exact test as indicated. Ordinal ranking scores were compared using the Mantel-Haenszel test.

## Results

One hundred twenty-eight charts were evaluated between January 2007 and January 2012. Included were 73 chemotherapy and 55 chemotherapy, plus GKRS, patients. The follow-up period was 24 to 49 months (mean 36.9 months). There were no statistically significant differences in patient demographics or histology diagnosis (Table 2).

**Table 2:** Patient Demographic and Perioperative Data Values are given as mean and standard deviation, with or without range in parentheses and 95% confidence interval in brackets. † Relationship between the number of positive cases and the total number of assessed patients.

Demographic and Perioperative Data			
Patient Parameters	Control	GK Group	P value
Number of Patients	73	55	NA
Age (years)	[58.1 ± 5.3]	[56.9 ± 3.3]	0.685
Sex			
Male	33 (70%)	26 (56%)	0.51
Female	40 (30%)	19 (44%)	
Body-mass index (kg/m^2^)	[24.1 ± 2.6]	[22.2 ± 3.1]	0.39
Preop Diagnosis			
Tobacco use	16	22	0.25
Pre-albumin (mg/dL)	[18.1 ± 1.3]	[19.1 ± 0.9]	0.347
Albumin (g/dL)	[3.4 ± 0.4]	[3.2 ± 0.4]	0.89†
Absolute Lymphocyte Count (cells/μL)	[1745 ± 468]	[1798 ± 401]	0.20
Median Survival	26.8 months (18.3 – 29.1)	47.6 months (28.3 – 49.1)	0.0034
Preop Diagnosis			0.59†

**Figure 1:**
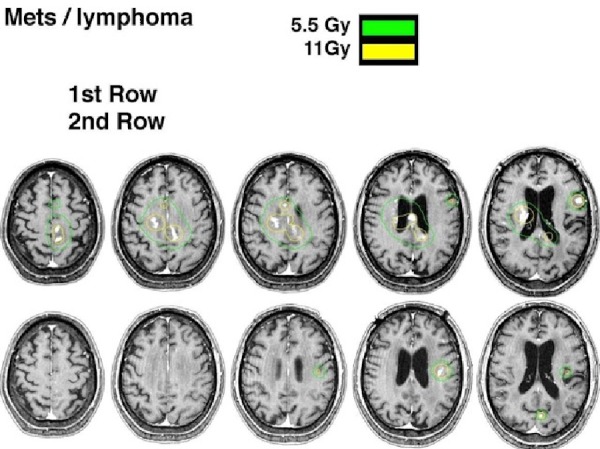
Brain MRI before and two months after GKRS treatment

The histologic finding in all patients was the presence of large, diffuse B-cell lymphoma. Patients were treated with GKRS doses ranging from 11 Gy to 16 Gy (median: 11 Gy) 50% isodose line (Figure 1).

The median survival rate from initial diagnosis was statistically significant (p = 0.0034) with 26.8 months in the chemotherapy group and 47.6 months in patients receiving chemotherapy, plus GKRS. The signs and symptoms of the patients remained evidently improved within two to six weeks after GKRS and four to 10 weeks after chemotherapy alone. Lesions treated with GKRS showed a complete response on MRI when evaluated three to eight weeks (mean range: 6.3 weeks) post-therapy.

The clinical data was gathered for brain tumor lesion control, disease control, toxicity, recurrence, and survival as seen in Table 3.

**Table 3:** Brain Tumor-Related Outcomes

Brain Tumor-Related Outcomes			
Patient Parameters	Control Group	Gamma Knife	P Value
Tumor Size			
Mean Size cm^3^	3.7	3.5	0.18
Median Size cm^3^	2.9	2.7	0.27
Size Range cm^3^	1.3 – 6.9	1.5 – 6.4	0.21
Presence of single or multiple lesions (%)			
Single	18%	22%	0.35
Multiple	92%	78%	0.28
Tumor location			
Frontal	10	12	0.12
Parietal	14	11	0.106
Temporal	19	14	0.09
Occipital	7	7	0.58
Cerebellum	6	4	0.31
Extracranial metastasis			
Yes	10	16	0.09
No	63	39	0.072
VP Shunt			
Yes	24	14	0.068
No	49	41	0.35
Prior cancer	11	6	0.24
Systemic symptoms	5	3	0.587
Positive CSF cytology examination	73	51	0.187
High CSF protein level	51	43	0.14
Multiple lesions	42	29	0.0687

The Karnofsky performance grade was enhanced from a preoperative average of 55% to a postoperative average of 88% in the GKRS group. Significant factors contributing to survival greater than 24 months following GKRS were increased marginal dose (odds ratio = 6.5, p = 0.031, 95% CI (1.4-18.57)), increase in maximal dose (odds ratio = 1.54, p = 0.012, 95% CI (1.15-2.89)), and increase in the pretreatment Karnofsky score (odds ratio = 5.13, p = 0.008, 95% CI (1.55-85.1)). The side effects attributed to GKRS were minimal; none of the patients had a deterioration related to the treatment. No complications were related after the procedure.

## Discussion

The dilemma faced in the treatment of PCNSL is the determination of a superior approach that will offer higher success rates while limiting adverse effects, such as neurotoxicity. While the gold standard therapy for patients with newly diagnosed PCNSL remains high-dose MTX, the use of higher doses of MTX regimens in elderly patients may be associated with a higher risk of systemic toxicity because of the increased prevalence of comorbidities [9]. Combination chemotherapy regimens, such as the methotrexate, cytarabine, thiotepa, and rituximab (MATRix) regimen, are associated with a greater amount of hematologic complications, including anemia, neutropenia, and thrombocytopenia [10]. Historically, there has been a high incidence of severe neurotoxicity in patients receiving whole brain radiotherapy (WBRT), predominantly those older than 65 years of age [2, 11-12]. Moreover, up to 35-50% of patients present with neurologic deterioration after WBRT, resulting in a five-year mortality rate of 30% [2, 13].

GKRS, in comparison to WBRT, provides targeted radiation to the lesion, perhaps attributing to a lower incidence of neurotoxicity. Of note, not a single patient experienced any mental or memory impairment in our study, which is in partial agreement with an earlier report [6]. Kenai, et al. described a mean recurrence-free period of 24.4 months for their patients [6]. In our observational study, there were 16 patients each with more than seven brain lesions, an association with poor clinical prognosis. Nine patients developed new lesions in other areas of the brain and repeated GKRS was performed. No local recurrence was observed in any patient at a mean of 30.6 months post-GKRS treatment. The median survival rate from initial diagnosis was statistically significant (p-value: 0.0034) with 26.8 months in the chemotherapy group and 47.6 months in patients receiving chemotherapy, plus GKRS. Therefore, our findings are in accordance with those of Kenai and seem to be at a higher success rate than those described by others in treating patients with chemotherapy and/or radiotherapy alone.

While there appeared to be a predisposition in the direction of new tumor growth in individuals who did not undergo GKRS treatment, we do not suggest a connection between recurrence and original appearance of multiple or solitary lesions, but current systemic chemotherapy would be essential. Overall, GKRS appears to be a valuable method for the management of primary and relapsed CNSL, since it enables exceptional local control in a short-term period without severe neurotoxicity or complications [14].

## Conclusions

GKRS is a minimally invasive procedure that allows for the immediate use of systemic chemotherapy and can be an ideal co-adjuvant treatment option in patients with PCNSL. The combination allows for greater tumor control, enhanced overall survival period, and a lesser number of complications.
